# Alternative neural circuitry that might be impaired in the development of Alzheimer disease

**DOI:** 10.3389/fnins.2015.00145

**Published:** 2015-04-23

**Authors:** Jesus Avila, George Perry, Bryan A. Strange, Felix Hernandez

**Affiliations:** ^1^Neurobiology, Centro de Biologia Molecular Severo Ochoa (CSIC-UAM)Madrid, Spain; ^2^Centro de Investigacion Biomedica en Red de Enfermedades NeurodegenerativasMadrid, Spain; ^3^College of Sciences, The University of Texas at San AntonioSan Antonio, TX, USA; ^4^Department of Neuroimaging, Reina Sofia Foundation, Center for Alzheimer Research, FCIENMadrid, Spain; ^5^Laboratory for Clinical Neuroscience, CTB, Universidad Politecnica de MadridMadrid, Spain

**Keywords:** Alzheimer disease (AD), cognitive decline, tau proteins, abeta, tauopathies

## Abstract

It is well established that some individuals with normal cognitive capacity have abundant senile plaques in their brains. It has been proposed that those individuals are resilient or have compensation factors to prevent cognitive decline. In this comment, we explore an alternative mechanism through which cognitive capacity is maintained. This mechanism could involve the impairment of alternative neural circuitry. Also, the proportion of molecules such as Aβ or tau protein present in different areas of the brain could be important.

## Introduction

**Figure 1:**
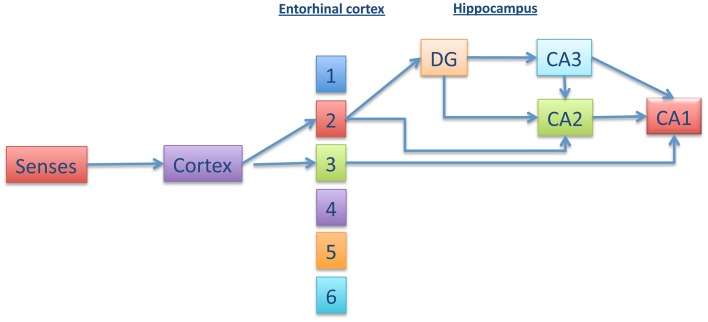
**Different pathways connecting entorhinal cortex with CA1**. Information from the senses is transmitted to the cortex and, afterwards, to the enthorhinal cortex (EC) (upper layers). From EC, the information could go directly to CA1 or, indirectly, through dentate gyrus (DG), CA3 (or CA2), and CA1. This trisynaptic pathway involves adult neurogenesis.

Loss of episodic memory is the most well known feature of Alzheimer disease (AD). Braak and Braak (1996), suggested that damage of the connections between the entorhinal cortex (EC) and hippocampal area could play an important role in the memory impairment of AD (Gomez-Isla et al., 1996). Within the hippocampus, other studies have suggested that the CA1 hippocampal subregion could be the minimal region that is required for acquisition of episodic memory (Zola-Morgan et al., 1986; Volpe et al., 1992; Tsien et al., 1996; Shimizu et al., 2000; Bendel et al., 2005; Buenz et al., 2006; Bueters et al., 2008). There are several works indicating the possible (and different) pathways that connect the EC with CA1 (for a review see Moser et al., 2014 and Figure 1). Some of these pathways go through the dentate gyrus, in which adult neurogenesis could be involved in the formation of new memories (Zola-Morgan et al., 1986; Deng et al., 2010).

Damage to the EC and hippocampal region in AD is associated with the appearance of senile plaques and neurofibrillary tangles, aberrant structures first described by Alzheimer (1907). Indeed, it is typically considered that a proper diagnosis of AD is only really complete when, at autopsy, the appearance of plaques and tangles are observed in the brain of the patient (Khachaturian, 1985).

However, the causal nature of the correlation between appearance of plaques and loss of memory (or cognitive impairment) is not clear. Thus, it has been proposed that the disease should be treated, not the lesions (Zhu et al., 2007), because the pathology may be a protective, possibly antioxidant response to the primary pathogenesis. Furthermore, studies on familial AD (FAD) cases reveal an asymptomatic phase in which there are plaques without cognitive impairment (Bateman et al., 2012), although it may also suggest that the presence of plaques is a first step that could favor the subsequent onset of the disease and that their presence is not immediately related to cognitive impairment (Bateman et al., 2012).

## Plaques and cognitive impairment

There is controversy surrounding a total correlation between the presence of plaques and the presence of cognitive impairment. Some time ago, Katzman et al described cases of cognitively normal people bearing plaques (Katzman et al., 1989). In a more recent study, analyzing those people without cognitive impairment, it was found that some people met the criteria for high likelihood AD, based on the presence of plaques (Bennett et al., 2006), indicating that AD pathology can be found in the brain of those without cognitive impairment (Aizenstein et al., 2008). Some functional magnetic resonance analysis have also reported the presence of Aβ aggregates in people without cognitive impairment (Dickerson et al., 2004; Sperling et al., 2009; Mormino et al., 2012).

More recently, Elman et al reported that some older people may maintain normal cognition despite the presence of plaques observed by positron emission tomography (PET) analyses (Elman et al., 2014). There are at least two additional explanations from those discussed above, a possible resilience, based on personal characteristics such as having a higher cognitive reserve (Xu et al., 2014), or compensation of a degenerated pathway by using an alternative functional pathway (Elman et al., 2014). In addition, it can be suggested that failure of more than one neuronal circuit can be needed for cognitive impairment.

This led us to consider another alternative to that involving the connections from EC to CA1, indicated in Figure 1, an alternative that might prevent cognitive decline.

In the work of Elman et al. (2014), there are explanations that involve different types of connections, based on fMRI studies, suggesting that CA1 can connect with the cortex without going through the EC. In those studies, Elman et al. (2014) took advantage of the system by analyzing two networks that were previously identified. One of them links those areas that respond to a specific cognitive activity, like visualization of a photograph (Fox et al., 2005; Xu et al., 2014). This is the task-positive network (TPN) (Fox et al., 2005). The other one indicates the regions that are activated in the resting state (Buckner et al., 2008). This is the default mode network (DMN). The DMN can be disrupted in some neurodegenerative disorders (Buckner et al., 2008).

In human subjects, with normal cognition and no plaques, engaging in a task activates the TPN while the DMN shuts down. However, Elman et al. (2014) found that in the same conditions, people with plaques and without cognitive impairment also displayed higher TPN activity but—critically—DMN was less deactivated. The authors focused on this decrease of DMN shut down to try to explain the maintenance of cognition. Indeed, there are several works, using fMRI analysis, or other image techniques, indicating the connection between dysfunction of DMN and cognitive decline. We include some examples of those works (Petrella et al., 2011; Wang et al., 2013; Garces et al., 2014; Gardini et al., 2015).

## Alternative pathways to CA1

**Figure 2:**
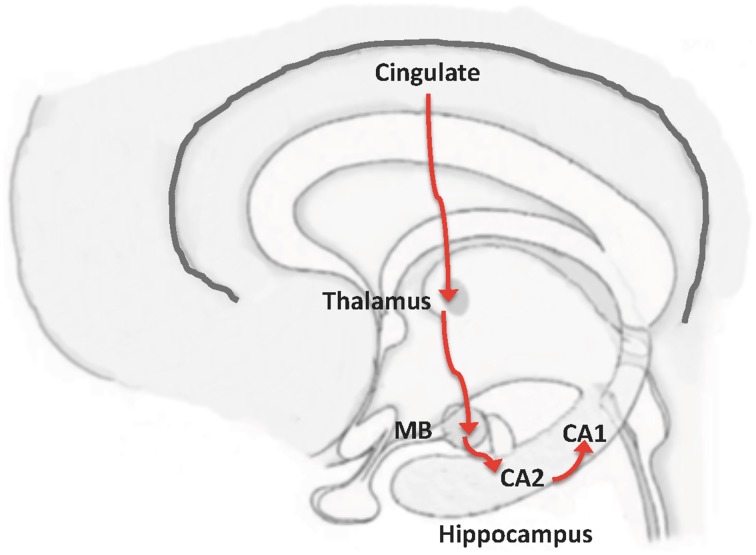
**From neocortex to hippocampus, by-passing entorhinal cortex: the MB-CA2-CA1 pathway**. A possible pathway to connect the cortex with CA1, without transmission through EC, could be based on a proposed connection between mammillary bodies (MB) to CA2, and from CA2 to CA1 (Haglund et al., 1984; Kohara et al., 2014). In this way, the previously known connection shown in the Figure could be complemented by the following pathway: Cortex 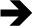 cingulum 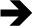 mammillary bodies 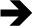 CA2 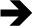 CA1.

The activated areas in DMN include prefrontal cortex and posterior cingulate cortex (Buckner et al., 2008). Cingulate cortex is connected to other structures involved in memory, including the mammillary bodies (MB) (Vann and Aggleton, 2004; Shah et al., 2012). A connection between MB and CA2, which in turn is connected to CA1, has also been found (Haglund et al., 1984; Kohara et al., 2014). Thus, we propose this MB-CA2-CA1 circuit as an alternative pathway through which information from the cerebral cortex can reach CA1 and avoid the EC (Figure 2).

We suggest that, for severe cognitive impairment to occur, disruption of both circuits, that involving EC-CA1 and that involving cingulate cortex-CA1, is needed. Indeed, abnormal connectivity between the posterior cingulate and hippocampus has been described in patients with cognitive impairment (Zhou et al., 2008). In addition, it has been also indicated that a decrease in the functional connectivity at the posterior cingulate cortex can be critical for the conversion from mild cognitive impairment to AD (Bozzali et al., 2014). Also, in the pioneering works of Stern (2002, 2006) on resilience (cognitive reserve) or compensation, the loss of connectivity at the posterior cingulate cortex was already proposed. In this comment, our main focus is not on resilience (see on that subject the recent work of Pereira et al., 2014), but on compensation.

If, indeed, there is first a loss of connectivity at the hippocampal area (location of the dentate gyrus), followed by a loss of connectivity at the posterior cingulate cortex (Zhou et al., 2008), risk factors, such as aging, could also play a role in this process. It has been recently reported that there is a brain network that links development, aging, and vulnerability to AD (Douaud et al., 2014). This link is based on the so-called Ribot's law indicating that the destruction of memories progresses in reverse order to that of their formation (Douaud et al., 2014). Brain structures, like dentate gyrus, that play a role in recent memory, are assembled very late in development but can lose their functionality very early in the neurodegeneration process.

We suggest that, it is possible that both mechanisms, that involving EC-CA1 and that involving posterior cingulate cortex-CA1, could be damaged and are needed to develop a severe cognitive impairment and AD. Thus, both mechanisms should be needed for disease progression.

## Distribution of Aβ and tau in cingulate gyrus and enthorinal cortex

As previously indicated, the brain of AD patients contain senile plaques, composed of amyloid-beta peptide (Aβ) and neurofibrillary tangles, containing tau protein polymers. By looking at the causes of FAD, genetic analyses have indicated that mutations in APP (amyloid precursor protein), PSEN-1 (presenilin 1) or PSEN-2 (presenilin 2) genes are the cause of the different types of familial disease. The main consequence of these mutations is an increased production of Aβ. This observation resulted in the proposed amyloid cascade hypothesis of AD. This hypothesis indicates that Aβ accumulation in brain is the primary factor driving AD pathology (Selkoe, 1991; Hardy and Higgins, 1992).

Afterwards, it was shown that the presence of tau protein is essential to the amyloid-beta induced neurotoxicity, occurring in AD (Rapoport et al., 2002; Roberson et al., 2007; Ittner et al., 2010). Based on these and other reports, it was suggested that Aβ could initiate the pathological process but the presence of tau is needed for the progression of the process. In this way, the cell co-localization of Aβ and tau could increase the possibility of neuronal damage.

At the molecular level, a double dissociation in regional distribution of tau and amyloid-beta has been reported when comparing cingulate gyrus and enthorhinal cortex in post-mortem Alzheimer's brains (Shukla and Bridges, 1999). It was described that tau load was almost twice as great in the enthorinal cortex than elsewhere in the brain, whereas Aβ levels were much higher in the cingulate gyrus compared to enthorinal cortex (Shukla and Bridges, 1999). It should be known if those differences may play a role in the development of the disease, mainly within the cingulate cortex, although we know that EC and posterior cingulate cortex could be interconnected and that modified tau could spread from EC to the cingulate cortex (Yassa, 2014). That transport time of modified tau from EC to posterior cingulate cortex may determine the progression time of the disease during the transition from MCI to AD.

In many transgenic animal models, there are no differences in the expression of tau or Aβ at specific locations of the brain. This is because, sometimes, the expression of tau or APP mRNAs is under strong promoters and it may facilitate the expression of a protein throughout the whole brain in a non-physiological way. Thus, in these animal models the progression of degeneration may take place in a different fashion than that occurring in the human disease.

If both, Aβ and tau aggregates, play a common, important role in the pathogenesis of AD, it will be of interest to determine the localization and overlap of those aggregates in different brain regions, given that the presence of plaques in the absence of tau, in some regions, might not be sufficient for cognitive decline. In this way, decreasing the amount of tau at specific brain regions could have a therapeutic function if, as indicated, the common presence of Aβ and tau aggregates is needed for the progression of the disease. Determining Aβ and tau overlap may, however, be complex as clear differences in the amount of tau can be found even between subregions of memory-related brain areas, e.g., hippocampus CA1 (high tau amount), vs. CA3 and CA2 (low tau amount) (our preliminary results). In addition, the presence (or absence) of other molecules related to Aβ or tau pathology could play a role in the development of neurodegeneration. A recent example of this is the telomerase protein TERT that apart from being protective against oxidative damage has a protective role against tau pathology (Spilsbury et al., 2015). Previously it was reported that a coordinated expression of tau and heme oxygenase 1 may play a pivotal role in the cytoprotection of neuronal cells (Takeda et al., 2004). The oxidative imbalance in AD has been extensively reviewed (Zhu et al., 2005; Mondragon-Rodriguez et al., 2013).

### Conflict of interest statement

The authors declare that the research was conducted in the absence of any commercial or financial relationships that could be construed as a potential conflict of interest.
